# Higher C-reactive protein to high-density lipoprotein cholesterol ratio is associated with hyperuricemia in diabetes and prediabetes: a cross-sectional study

**DOI:** 10.3389/fendo.2025.1619370

**Published:** 2025-06-27

**Authors:** Dongni Huang, Jing Ma, Yan Zhao, Qi Pan, Guogang Xu, Lixin Guo

**Affiliations:** ^1^ Department of Endocrinology, Beijing Hospital, National Center for Gerontology, Institute of Geriatric Medicine, Chinese Academy of Medical Sciences, Beijing, China; ^2^ Graduate School of Peking Union Medical College, Chinese Academy of Medical Sciences, Beijing, China; ^3^ Health Management Institute, The Second Medical Center & National Clinical Research Center for Geriatric Diseases, Chinese PLA General Hospital, Beijing, China

**Keywords:** hyperuricemia, CRP/HDL-c ratio, diabetes, prediabetes, inflammation, lipid metabolism

## Abstract

**Background:**

The ratio of C-reactive protein to high-density lipoprotein cholesterol (CRP/HDL-c) reflects systemic inflammation and lipid status, both of which are implicated in uric acid metabolism. This study aimed to investigate the association between CRP/HDL-c and the prevalence of hyperuricemia (HUA) among adults with diabetes or prediabetes.

**Methods:**

This cross-sectional study included 10915 adults with diabetes or prediabetes from the Health Management Institute of the PLA General Hospital. Hyperuricemia was defined as a serum uric acid concentration ≥7 mg/dL in men and ≥6 mg/dL in women. Participants were divided into quartiles according to the ratio. Multivariate logistic regression and restricted cubic spline analyses were used to assess associations. Subgroup analyses and interaction tests were performed.

**Results:**

The prevalence of HUA increased across CRP/HDL-c quartiles (18.43%, 20.39%, 24.54%, and 29.82%; P < 0.001). Higher CRP/HDL-c levels were independently associated with increased HUA risk (odds ratio [OR] = 1.64, 95% confidence interval [CI]: 1.14–2.36; *P* = 0.008). Participants in the highest quartile had a significantly higher risk compared to those in the lowest quartile (OR = 1.33, 95% CI: 1.15–1.54; *P* < 0.001). The association was stronger in females (OR = 1.30) than in males (OR = 1.14), with a significant gender interaction (*P* for interaction = 0.031). Among females, the association was more pronounced in those aged <50 years (OR = 1.47). RCS analysis indicated a linear dose–response relationship.

**Conclusions:**

An elevated C-reactive protein to high-density lipoprotein cholesterol ratio is significantly associated with a higher risk of hyperuricemia in adults with diabetes or prediabetes, particularly in younger females.

## 1 Introduction

Hyperuricemia (HUA) is a metabolic disorder syndrome caused by abnormalities in purine metabolism. Uric acid, the end product of purine metabolism, is mainly produced in the liver. Under normal conditions, uric acid production and excretion are maintained in dynamic balance. When uric acid production increases excessively or excretion decreases, serum uric acid levels rise, leading to HUA. Epidemiological and clinical studies have linked HUA with the development of various diseases, including chronic kidney disease, fatty liver, metabolic syndrome, hypertension, insulin resistance, obesity, as well as cardiovascular and cerebrovascular diseases (1–3). It is also an independent predictor of premature death (4). Recent trends suggest an increase in the prevalence of HUA, which is attributed to changes in lifestyle, particularly in high- and middle-income countries (5). According to the National Health and Nutrition Examination Survey (NHANES) in the United States, approximately 21% of adults, or 43 million people, have been diagnosed with HUA (6). The prevalence of HUA in China and South Korea is 6.4% and 11.4%, respectively (7, 8). HUA prevalence among diabetic patients has been reported to be 21.24% in China (9) and 20.70% in North America (10).

HUA is closely associated with abnormal glucose metabolism (11). Individuals with elevated serum uric acid levels are significantly more likely to develop diabetes than those with normal levels, and conversely, people with diabetes have an increased risk of developing HUA (12, 13). This bidirectional relationship likely involves multiple mechanisms. HUA may disrupt glucose homeostasis by impairing insulin signaling and promoting insulin resistance (14). In parallel, diabetes is often accompanied by metabolic disturbances such as obesity and dyslipidemia, which can further exacerbate uric acid metabolism disorders (15). Therefore, managing serum uric acid levels in patients with diabetes or prediabetes is essential—not only to reduce the risk of gout, but also to improve overall metabolic health, lower the incidence of chronic complications, and enhance both quality of life and life expectancy.

HUA and abnormal glucose metabolism are both closely linked to inflammation (16, 17). Raised uric acid levels and chronic disturbances in glucose metabolism can activate intracellular signaling pathways and promote the expression of inflammatory cytokines, triggering systemic inflammation and contributing to organ damage. Inflammatory states, in turn, may increase the risk of HUA, diabetes, and diabetes-related complications, creating a self-reinforcing cycle. C-reactive protein (CRP) is a widely used inflammatory marker. It plays a key role in inflammatory responses, atherosclerosis, autoimmune conditions and cardiovascular disease (18–20). Dyslipidemia—characterized by elevated triglycerides, total cholesterol, low-density lipoprotein cholesterol, and reduced high-density lipoprotein cholesterol (HDL-c)—is associated with higher risks of diabetes and HUA (21). Low HDL-c, in particular, is a known risk factor for diabetes, cardiovascular disease, and other metabolic disorders (22, 23). The CRP/HDL-c has been proposed as a combined marker of inflammation and lipid status, and has shown associations with cardiovascular and metabolic disease risk (24, 25). One study found a significant association between CRP/HDL-c and HUA in US adults (26). However, no studies have yet examined this relationship in people with diabetes or prediabetes, highlighting a gap that warrants further investigation.

Using data sourced from the Health Management Institute of the Second Medical Centre, PLA General Hospital, the aim of this study was to investigate the relationship between the CRP/HDL-c ratio and the risk of developing HUA in this population. The hypothesis of this study was that there would be a strong correlation between the CRP/HDL-c ratio and the risk of HUA among individuals with diabetes or prediabetes.

## 2 Materials and methods

### 2.1 Study population

This study utilized data from the Health Management Research Institute of PLA General Hospital, collected between November 2009 and December 2016. A total of 10,915 individuals aged 18 to 80 years with either diabetes or prediabetes were retrospectively included. All participants provided written informed consent for the use of their clinical data at the time of their hospital visit, in line with institutional policies. The study was approved by the Research Ethics Committee of Beijing Hospital (2025BJYYEC-KY03901). Diabetes and prediabetes were defined according to the American Diabetes Association (ADA) criteria (27). Diabetes was diagnosed based on a self-reported history of physician-diagnosed diabetes, a fasting plasma glucose (FPG) level ≥126 mg/dL, a 2-hour plasma glucose level ≥200 mg/dL after a 75-g oral glucose tolerance test (OGTT), or a hemoglobin A1c (HbA1c) level ≥6.5%. Prediabetes was defined as the absence of a prior diabetes diagnosis combined with either impaired fasting glucose (FPG 100–125 mg/dL), impaired glucose tolerance (2-hour plasma glucose 140–199 mg/dL after OGTT), or an HbA1c level of 5.7–6.4%. FPG was measured after an overnight fast of at least 10 hours. Both FPG and 2-hour plasma glucose levels following the OGTT were assessed according to standardized procedures. HbA1c was measured in a central laboratory using high-performance liquid chromatography with the boronate affinity method (Bio-Rad D-10 Hemoglobin Analyzer) following established protocols. Exclusion criteria included: (1) missing CRP or HDL-c data; (2) age <18 years; (3) pregnancy; (4) acute inflammatory or infectious diseases at the time of data collection; (5) use of medications influencing CRP or HDL-c (e.g., corticosteroids, statins); and (6) diagnosed malignancies.

### 2.2 Exposure and outcome definitions

In this study, the CRP/HDL-c, which is the ratio of CRP to HDL-c, was used as the exposure variable. HUA was defined as SUA levels ≥ 7 mg/dL (420umol/l) in men and ≥ 6 mg/dL(360umol/l) in women (28).

### 2.3 Covariate definitions

The study accounted for multiple potential covariates, including age (years), gender, smoking status, diabetes mellitus status, hypertension status, blood pressure, body mass index (BMI, kg/m²), waist-to-hip ratio (WHR), and a series of biochemical markers. These included glycated hemoglobin (HbA1c, %), alanine aminotransferase (ALT, U/L), aspartate aminotransferase (AST, U/L), triglycerides (TG, mmol/L), low-density lipoprotein cholesterol (LDL-C, mmol/L), creatinine (Cr, µmol/L), and estimated glomerular filtration rate (eGFR, mL/min/1.73 m²). BMI was categorized as <25 kg/m² (normal), 25–29.9 kg/m² (overweight), or ≥30 kg/m² (obese). The eGFR was calculated using the Chronic Kidney Disease Epidemiology Collaboration (CKD-EPI) equation (29). Smoke was defined as the consumption of ≥10 cigarettes per day for ≥1 year, based on criteria from relevant literature (30). Alcohol consumption was classified as limited drinking (no alcohol or ≤25 g/day for men and ≤15 g/day for women) or excessive drinking (≥25 g/day for men and ≥15 g/day for women) (31). Hypertension status was determined based on self-reported history.

### 2.4 Data collection

Electronic medical records were retrospectively reviewed to obtain demographic and biochemical data. Information including age, systolic and diastolic blood pressure (SBP and DBP), smoking status, and alcohol consumption was collected using standardized methods. Fasting venous blood samples were drawn from all participants following an overnight fast and processed in accordance with the quality control standards of the Clinical Laboratory at PLA General Hospital (32). Serum concentrations of total cholesterol (TC), triglycerides (TG), high-density lipoprotein cholesterol (HDL-C), low-density lipoprotein cholesterol (LDL-C), fasting blood glucose (FBG), hemoglobin A1c (HbA1c), alanine aminotransferase (ALT), aspartate aminotransferase (AST), blood urea nitrogen (BUN), creatinine (Cr), and uric acid (UA) were measured using validated laboratory techniques. C-reactive protein (CRP) levels were determined by nephelometry. The CRP-to-HDL-C ratio was subsequently calculated.

### 2.5 Statistical analysis

All statistical analyses were performed using R software (version 4.4.3). Continuous variables with a normal distribution were expressed as mean ± standard deviation (SD), while those with a skewed distribution were presented as median (interquartile range). Group differences in continuous variables were assessed using the independent samples t-test or the Mann–Whitney U test, as appropriate. Categorical variables were compared using the chi-square test. Logistic and linear regression models were employed to evaluate the associations of CRP, HDL-C, and the CRP/HDL-C ratio with both the risk of HUA and SUA concentrations. Multicollinearity was assessed using the variance inflation factor (VIF). Decision curve analysis (DCA) and receiver operating characteristic (ROC) curve analysis were conducted to assess the predictive value of CRP, HDL-C, and the CRP/HDL-C ratio for identifying HUA risk. Subgroup analyses were also performed. Finally, restricted cubic spline (RCS) logistic regression with three knots was applied to explore potential nonlinear relationships between the CRP/HDL-C ratio and the risk of developing HUA. A two-sided P value < 0.05 was considered statistically significant.

## 3 Results

### 3.1 Baseline characteristics of study participants

**Table 1** showed the baseline characteristics of the study participants (n = 10915) with diabetes or pre-diabetes stratified by without or with hyperuricemia. The average age of the participants was 51.63 years, and 72.23% of them were male. Compared with the non-HUA group, the HUA group was younger and had more males, drinkers, and individuals with diabetes (P < 0.05). Additionally, individuals in this group exhibited increased SBP, DBP, BMI, WHR, ALT concentrations, AST concentrations, BUN concentrations, Cr concentrations, and UA concentrations, FBG values, FCP concentrations, FINS concentrations, HOMA-IR, TG concentrations, TC concentrations, LDL-c concentrations, CRP (P < 0.001). Conversely, reduced eGFR and lower levels of HDL-c were significantly more common in the HUA group (P < 0.001). The HUA group exhibited notably greater CRP/HDL-c values than did the non-HUA group (P < 0.001).

**Table 1:** Characteristics of participants with diabetes or pre-diabetes stratified by without or with hyperuricemia.

Characteristic	Non-HUA group (N = 8,372)	HUA group (N = 2,543)	*p*-value
Age (years)	51 (47, 57)	50 (45, 55)	<0.001
Male gender, n (%)	5,694 (68%)	2,190 (86%)	<0.001
SBP (mmHg)	123 (112, 135)	127 (116, 138)	<0.001
DBP (mmHg)	79 (72, 87)	82 (76, 89)	<0.001
BMI (kg/m^2^)	25.6 (23.7, 27.7)	27.0 (25.1, 29.0)	<0.001
WHR	0.93 (0.86, 0.97)	0.95 (0.92, 0.98)	<0.001
ALT (U/L)	21 (15, 30)	26 (18, 38)	<0.001
AST (U/L)	19 (16, 23)	20 (17, 26)	<0.001
BUN (mmol/l)	5.10 (4.40, 6.00)	5.30 (4.50, 6.20)	<0.001
Cr (umol/l)	67 (57, 76)	75 (65, 83)	<0.001
eGFR(mL/min/1.73 m2)	103 (96, 109)	101 (92, 108)	<0.001
SUA (umol/l)	325 (276, 367)	455 (432, 494)	<0.001
FBG (mmol/l)	5.76 (5.24, 6.77)	5.87 (5.37, 6.64)	<0.001
HbA1c (%)	6.00 (5.80, 6.50)	6.00 (5.80, 6.40)	0.084
FCP (ng/ml)	2.52 (1.97, 3.20)	3.11 (2.57, 3.82)	<0.001
FINS (mU/L)	10 (7, 15)	12 (9, 17)	<0.001
HOMA-IR	2.62(1.73-3.93)	3.27(2.29-4.77)	<0.001
TG (mmol/l)	1.55 (1.10, 2.23)	2.08 (1.48, 3.08)	<0.001
TC (mmol/l)	4.80 (4.16, 5.47)	4.99 (4.35, 5.69)	<0.001
LDL-c (mmol/l)	3.15 (2.56, 3.73)	3.23 (2.62, 3.84)	<0.001
HDL-c (mmol/l)	1.15 (0.96, 1.38)	1.05 (0.90, 1.24)	<0.001
CRP (mg/l)	0.12 (0.07, 0.22)	0.15 (0.08, 0.27)	<0.001
CRP/HDL-c	0.11 (0.06, 0.20)	0.14 (0.07, 0.26)	<0.001
Smokers, n (%)	2,083 (25%)	661 (26%)	0.300
Drinkers, n (%)	2,595 (31%)	839 (33%)	0.013
Hypertension, n (%)	660 (7.9%)	204 (8.0%)	0.911
Diabetes, n (%)	1842 (22%)	786 (27%)	<0.001

Data are number of subjects (percentage) or medians (interquartile range).

Mann–Whitney U test was used to compare the median values between participants with and without hyperuricemia. Chi-square test was used to compare the percentage between participants with and without hyperuricemia.

SBP, systolic blood pressure; DBP, diastolic blood pressure; BMI, body mass index; WHR, waist to hip ratio; ALT, alanine aminotransferase; AST, aspartate aminotransferase; BUN, blood urea nitrogen; eGFR, estimated glomerular filtration rate; SUA, serum uric acid; FBG fasting blood glucose; HbA1c, glycated hemoglobin A; FCP, fasting plasma C-peptide; FINS, fasting insulin; HOMA-IR, homeostatic model assessment of insulin resistance; TG, triglycerides; TC, total cholesterol; LDL-c, low-density lipoprotein cholesterol; HDL-c, high- -density lipoprotein cholesterol.

### 3.2 Baseline characteristics based on the quantiles of the CRP/HDL-c

According to the CRP/HDL-c, the subjects were categorized into four groups based on quantiles (**Table 2**). The high-CRP/HDL-c quantile group exhibited greater proportions of males, individuals with diabetes and hypertension (P < 0.001). Furthermore, SBP, DBP, BMI, waist to hip ratio, ALT concentrations, AST concentrations, eGFR, FBG values, HbA1c values, FCP concentrations, FINS concentrations, HOMA-IR, TG concentrations, CRP were obviously increased (P < 0.001). In contrast, the proportions of individuals with high eGFRs, and high HDL-c levels decreased (P < 0.001). Compared to those in the lowest CRP/HDL-c quartile, individuals in the second, third and fourth quartile were younger (P < 0.001). Notably, a higher CRP/HDL-c was related to increased levels of SUA and a greater prevalence of HUA (18% vs.20% vs. 25% vs. 30%, P < 0.001).

**Table 2:** Baseline characteristics of participants according to the quartiles of the CRP/HDL-c.

Characteristic	Q1(0.02, 0.05) N = 2,729	Q2(0.07, 0.10) N = 2,731	Q3(0.13, 0.18) N = 2,726	Q4(0.27, 0.41) N = 2,729	*p* value
Age (years)	52 (47, 57)	51 (46, 56)	51 (46, 56)	50 (45, 56)	<0.001
Male gender, n (%)	1,826 (67%)	1,915 (70%)	2,021 (74%)	2,122 (78%)	<0.001
SBP (mmHg)	123 (111, 135)	122 (111, 135)	125 (113, 136)	127 (115, 138)	<0.001
DBP (mmHg)	80 (73, 87)	79 (72, 86)	80 (73, 87)	81 (74, 89)	<0.001
BMI (kg/m^2^)	24.9 (22.8, 26.8)	25.5 (23.6, 27.5)	26.3 (24.6, 28.4)	27.2 (25.2, 29.3)	<0.001
Waist to hip ratio	0.92 (0.84, 0.96)	0.93 (0.86, 0.96)	0.94 (0.89, 0.98)	0.95 (0.91, 0.99)	<0.001
ALT (U/L)	20 (15, 29)	21 (15, 30)	23 (17, 35)	24 (17, 36)	<0.001
AST (U/L)	19 (16, 23)	19 (16, 23)	19 (16, 24)	19 (16, 25)	<0.001
BUN (mmol/l)	5.20 (4.40, 6.09)	5.10 (4.40, 6.00)	5.20 (4.40, 6.10)	5.10 (4.40, 6.00)	0.200
Cr (umol/l)	69 (59, 79)	68 (58, 78)	68 (59, 78)	69 (59, 78)	0.400
eGFR (mL/min/1.73 m2)	104 (95, 110)	103 (96, 109)	102 (95, 109)	101 (94, 107)	<0.001
SUA (umol/l)	336 (278, 394)	341 (285, 399)	356 (301, 412)	367 (311, 425)	<0.001
FBG (mmol/l)	5.70 (5.21, 6.53)	5.68 (5.21, 6.57)	5.84 (5.32, 6.74)	5.94 (5.35, 7.13)	<0.001
HbA1c (%)	5.90 (5.80, 6.30)	6.00 (5.80, 6.40)	6.00 (5.80, 6.50)	6.10 (5.80, 6.80)	<0.001
FCP (ng/ml)	2.33 (1.81, 2.98)	2.51 (1.99, 3.13)	2.79 (2.23, 3.45)	3.03 (2.43, 3.76)	<0.001
FINS (mU/L)	9 (6, 13)	10 (7, 14)	11 (8, 16)	13 (9, 18)	<0.001
HOMA-IR	2.28 (1.52, 3.37)	2.53 (1.69, 3.71)	3.01 (2.05, 4.28)	3.38 (2.28, 5.15)	<0.001
TG (mmol/l)	1.49 (1.05, 2.23)	1.49 (1.06, 2.18)	1.72 (1.26, 2.41)	1.92 (1.38, 2.79)	<0.001
TC (mmol/l)	4.88 (4.19, 5.59)	4.83 (4.18, 5.47)	4.86 (4.24, 5.51)	4.81 (4.18, 5.50)	0.110
LDL-c (mmol/l)	3.18 (2.53, 3.82)	3.18 (2.59, 3.76)	3.20 (2.63, 3.75)	3.12 (2.56, 3.73)	0.072
HDL-c (mmol/l)	1.29 (1.07, 1.54)	1.19 (1.02, 1.39)	1.08 (0.92, 1.25)	0.98 (0.84, 1.14)	<0.001
CRP (mg/l)	0.04 (0.02, 0.06)	0.10 (0.08, 0.12)	0.17 (0.14, 0.20)	0.37 (0.27, 0.41)	<0.001
Smoke, n (%)	677 (25%)	708 (26%)	662 (24%)	697 (26%)	0.501
Drink, n (%)	881 (32%)	890 (33%)	899 (33%)	890 (33%)	0.988
Hypertension, n (%)	141 (5.2%)	232 (8.5%)	247 (9.1%)	253 (9.3%)	<0.001
Diabetes, n (%)	595 (22%)	604 (22%)	721 (26%)	900 (33%)	<0.001
HUA	503 (18.43%)	557 (20.39%)	669 (24.54%)	814 (29.82%)	<0.001

Data are number of subjects (percentage) or medians (interquartile ranges).

Kruskal-Wallis rank sum test was used to compare the median values between participants.

Chi-square test was used to compare the percentage between participants.

### 3.3 Association between the CRP/HDL-c and the risk of developing HUA

**Figure 1:**
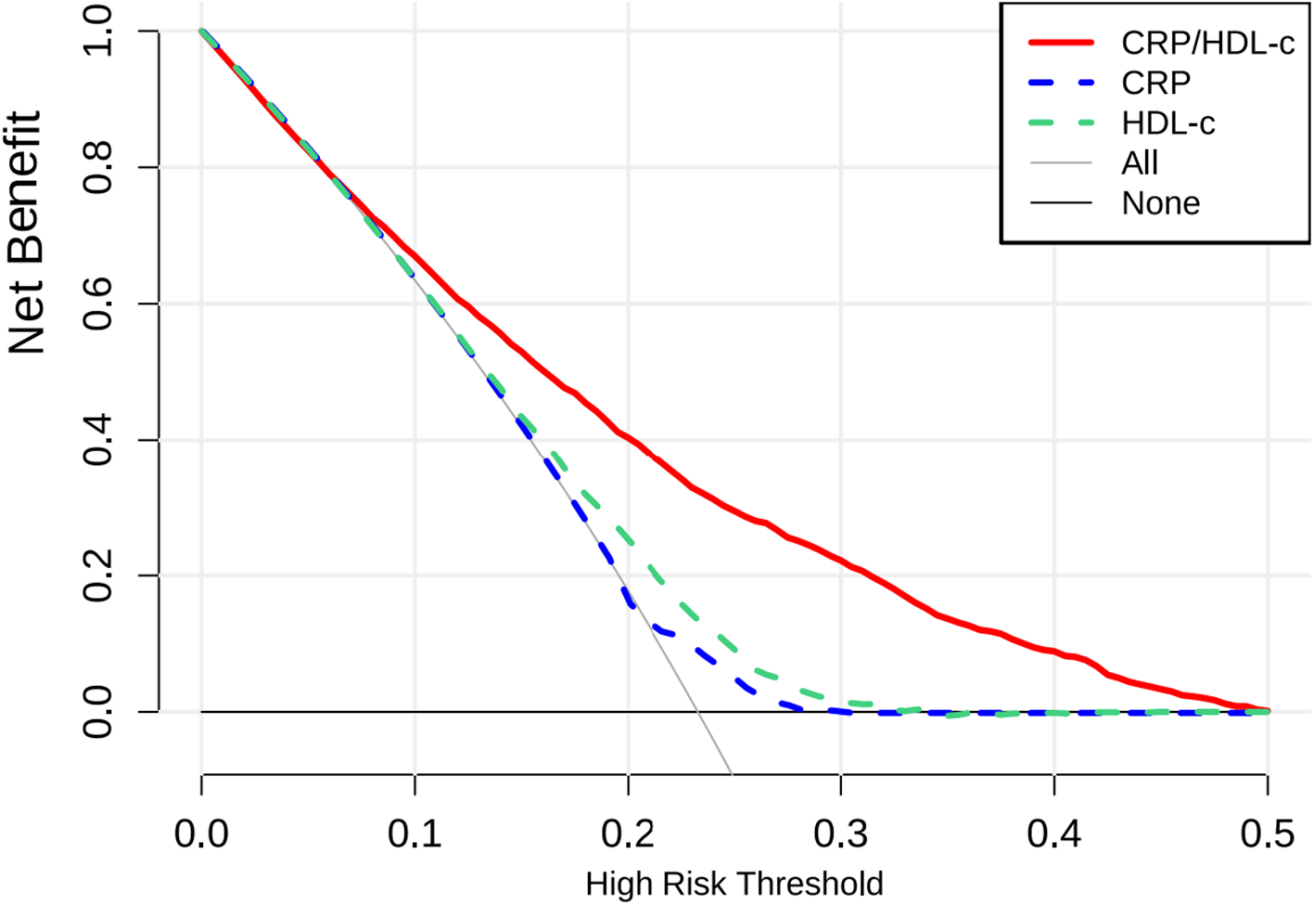
DCA results.

**Figure 2:**
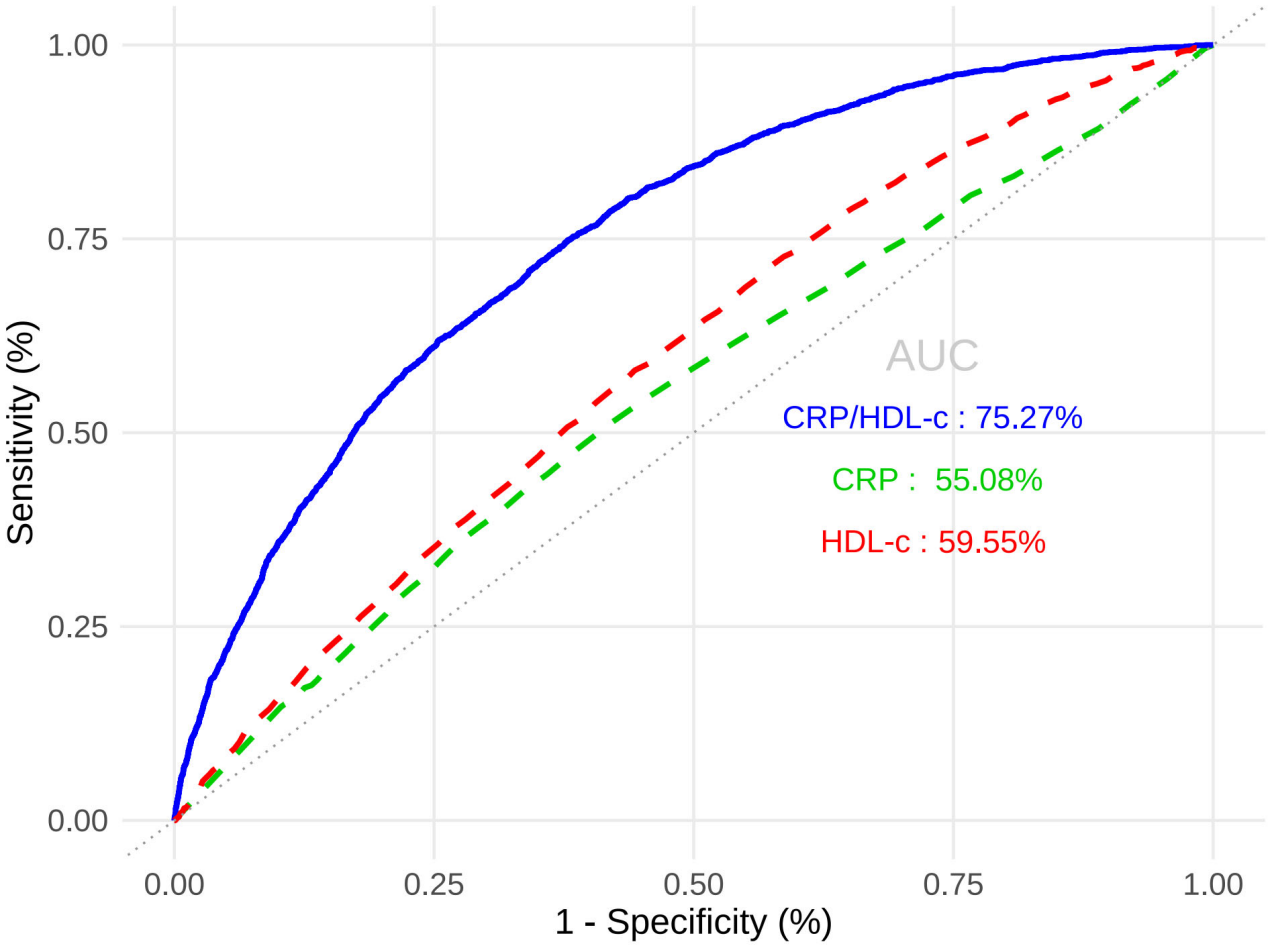
ROC results.

**Table 3A** showed that CRP/HDL-c was positively correlated with the prevalence of HUA, a statistically significant relationship that persisted across unadjusted, partially adjusted, and fully adjusted logistic regression models. After full adjustment, each unit increase in the CRP/HDL-c ratio was associated with a 64% higher risk of HUA (OR=1.64, 95% CI: 1.14 to 2.36; P=0.008; **Supplementary Tables 1**, **2**). When stratified by quartiles, participants in the highest quartile of CRP/HDL-c had a significantly greater risk compared with those in the lowest quartile (OR=1.33, 95% CI: 1.15 to 1.54; P<0.001).In a linear regression analysis using SUA as the dependent variable, CRP/HDL-c was positively and significantly associated with SUA levels (β=0.26, 95% CI: 0.10 to 0.43; P=0.002; **Table 3B**). Receiver operating characteristic (ROC) curve analysis showed that the areas under the curve (AUCs) for CRP/HDL-c, CRP, and HDL-c were 60.36%, 55.08%, and 59.55%, respectively. Decision curve analysis (DCA) further demonstrated that CRP/HDL-c provided a greater net benefit compared with CRP or HDL-c alone (**Figures 1**, **2**).

**Table 3:** Regression analyses for the association between CRP/HDL-c and HUA risk or SUA concentration.

A						
HUA	Model 1		Model 2		Model 3	
	OR (95%CI)	*P* value	OR (95%CI)	*P* value	OR (95%CI)	*P* value
Continuous						
CRP	2.25(1.55,3.29)	<0.001	2.24(1.54,3.25)	<0.001	1.95(1.26,2.98)	<0.001
HDL-c	0.30(0.26,0.36)	<0.001	0.63(0.53,0.75)	<0.001	0.90(0.71,1.14)	0.384
CRP/HDL-c	2.64(1.65,3.73)	<0.001	2.25(1.57,3.14)	<0.001	1.64(1.14,2.36)	0.008
Categories						
Quantile 1	reference		reference		reference	
Quantile 2	1.13(0.99,1.30)	0.067	1.02(0.89,1.17)	0.746	1.11(0.96,1.29)	0.146
Quantile 3	1.44(1.26,1.64)	<0.001	1.14(1.00,1.31)	0.049	1.17(1.02,1.36)	0.028
Quantile 4	1.88(1.66, 2.14)	<0.001	1.34(1.17,1.53)	<0.001	1.33(1.15,1.54)	<0.001
P for trend	< 0.001		<0.001		<0.001	
B						
SUA	Model 1		Model 2		Model 3	
	β(95%CI)	*P* value	β(95%CI)	*P* value	β(95%CI)	*P* value
Continuous						
CRP	1.00(0.81, 1.20)	<0.001	0.47(0.28,0.66)	<0.001	0.48(0.30,0.65)	<0.001
HDL-c	-1.30(-1.40, -1.20)	<0.001	-0.25(-0.33, -0.17)	<0.001	0.01(-0.11,0.09)	0.828
CRP/HDL-c	1.30(1.10, 1.50)	<0.001	0.47(0.30,0.65)	<0.001	0.26(0.10,0.43)	0.002
Categories						
Quantile 1	reference		reference		reference	
Quantile 2	0.10(0.02, 0.17)	0.009	-0.02(-0.08,0.05)	0.643	0.03(-0.03,0.09)	0.323
Quantile 3	0.35(0.27, 0.42)	<0.001	0.09(0.03,0.16)	0.004	0.10(0.04,0.16)	0.001
Quantile 4	0.53(0.46, 0.61)	<0.001	0.16(0.09,0.22)	<0.001	0.14(0.08,0.20)	<0.001
P for trend	< 0.001		< 0.001		< 0.001	

(A) Logistic regression analysis results for the association between the CRP/HDL-c and the risk of developing HUA. (B) Linear regression analysis results for the association between the CRP/HDL-c and the SUA concentration.

OR: odds ratio;95% CI: 95% confidence interval.

Model 2: adjusted for age, gender, BMI; Model 3: adjusted for age, gender, SBP, DBP, BMI, Waist to hip ratio, ALT, AST, BUN, Cr, eGFR, FBG, FCP, FINS, TC, TG, LDL-c, HbA1c, Drinkers, Hypertension.

### 3.4 Sensitivity analysis of individual CRP and HDL-c levels

To assess whether the observed association between the CRP/HDL-c ratio and HUA was driven primarily by changes in CRP or HDL-c alone, we conducted a sensitivity analysis evaluating the associations of CRP and HDL-c quartiles separately with HUA. As shown in **Supplementary Table 3**, compared with participants in the lowest CRP quartile, those in the third and fourth quartiles had significantly higher odds of hyperuricemia (Q3: OR=1.17, 95% CI: 1.01 to 1.34, P = 0.031; Q4: OR = 1.26, 95% CI: 1.10 to 1.45, P=0.009), indicating a positive association between systemic inflammation and uric acid levels. In contrast, higher HDL-c levels were inversely associated with hyperuricemia risk. Compared with the lowest HDL-c quartile, participants in the third and fourth quartiles exhibited significantly lower odds of HUA (Q3: OR=0.87, 95% CI: 0.73 to 0.98, P=0.045; Q4: OR=0.83, 95% CI: 0.68 to 0.95, P=0.032).These findings suggest that both elevated CRP and reduced HDL-c independently contribute to increased HUA risk, thereby supporting the use of CRP/HDL-c as an integrative biomarker reflecting combined inflammatory and lipid-related risk factors.

### 3.5 Subgroup analysis

**Figure 3:**
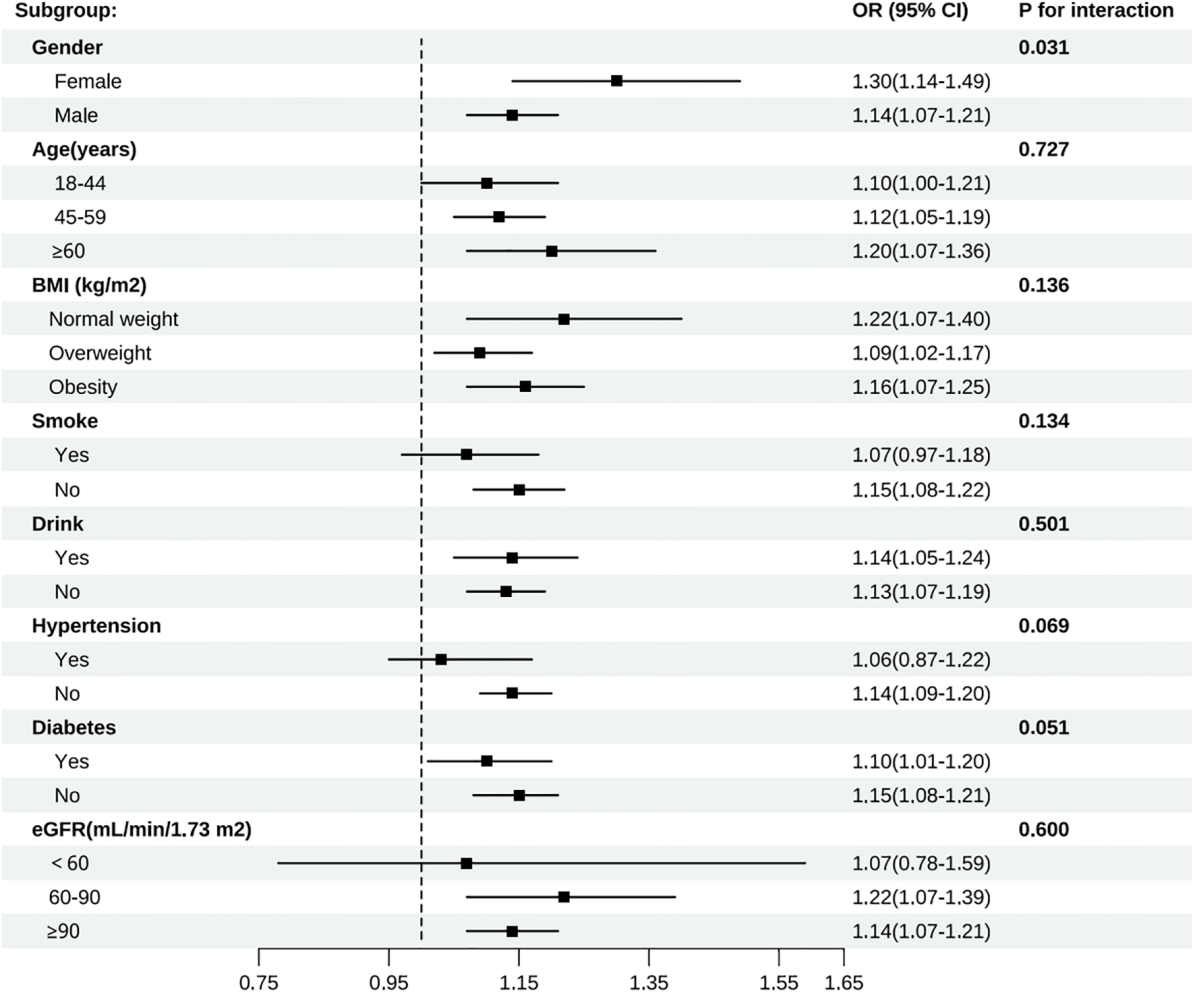
Subgroup analyses of all participants by gender, age, BMI, smoke, drink, hypertension, diabetes, and eGFR group.

Subgroup analyses were carried out considering factors such as age, gender, BMI, smoking status, drinking status, diabetes status, hypertension status, and eGFR to evaluate the robustness of the association between CRP/HDL-c and the risk of developing HUA across different populations with diabetes or pre-diabetes (**Figure 3**). The results indicated that the positive association remained consistent across most subgroups. Notably, the association was stronger in females (OR = 1.30, 95% CI: 1.14–1.49) than in males (OR = 1.14, 95% CI: 1.07–1.21), with a significant interaction by gender (P for interaction = 0.031). Further age-stratified analyses among females revealed that the OR for the <50 years group was 1.47, which was higher than the OR of 1.23 observed in the ≥50 years group (P for interaction = 0.008) (**Supplementary Figure 1**). This finding suggests that the CRP/HDL ratio may serve as a stronger predictor of hyperuricemia in younger women. No significant interactions were observed for age, BMI, smoking status, drinking status, diabetes, hypertension, or eGFR (all P for interaction > 0.05). Furthermore, the RCS results showed a linear relationship between the CRP/HDL-c and the risk of developing HUA across the entire diabetes and prediabetes population (**Supplementary Figure 2**).

## 4 Discussion

This population-based study provides novel evidence for an association between the CRP/HDL-c ratio and the risk of HUA in patients with diabetes or prediabetes. Compared with traditional lipid and inflammatory markers, a higher CRP/HDL-c ratio was more strongly associated with an increased risk of HUA in patients with diabetes or prediabetes.

Currently, an estimated 537 million adults globally are affected by diabetes, with over 90% of cases classified as type 2 diabetes mellitus (T2DM). This number is projected to increase to 783 million by 2045 (33). HUA and T2DM commonly coexist, and individuals with HUA have a 1.5-fold higher risk of developing T2DM compared with the general population (13). A cross-sectional analysis involving 1,037 individuals with diabetes and 272 healthy controls reported a significantly higher prevalence of HUA among patients with diabetes than among controls (34). Other studies have similarly shown that the prevalence of HUA is highest among individuals with impaired glucose tolerance, exceeding that observed in patients with T2DM or prediabetes individuals (35). Elevated serum uric acid levels have also been linked to an increased risk of chronic kidney disease (36), cardiovascular disease, hypertension (37), T2DM, metabolic syndrome (38, 39), and cognitive decline (40). Although the causal relationship between HUA and diabetes-related complications remains uncertain, several studies have identified elevated UA levels as a risk factor for atherosclerosis in patients with T2DM (41, 42). Given these findings, greater attention should be paid to the management of serum UA levels in individuals with diabetes or prediabetes to prevent the development of HUA. The identification of novel predictive markers is also warranted to enable early detection and timely intervention.

CRP/HDL-c has been introduced into clinical practice as a potential marker for predicting metabolic syndrome (MetS) (43). Compared with single biomarkers, this ratio may better capture the interplay between lipid metabolism and systemic inflammation, particularly in individuals with diabetes or prediabetes, thereby improving the accuracy of clinical risk stratification. In our study, the CRP/HDL-C ratio was strongly associated with the risk of HUA in both diabetic and prediabetic populations. Insulin resistance (IR), a common feature of diabetes and prediabetes, contributes to hyperglycemia, which may increase hepatic gluconeogenesis, impair lipid metabolism, and promote oxidative stress and inflammation—mechanisms that are likely to underlie the development of HUA (44). Previous animal studies have shown that small-molecule inhibitors targeting fatty acid synthase can improve hepatic function, reduce inflammation, and attenuate oxidative stress in obese mice fed a high-sugar diet (44).Our findings also showed that patients with diabetes or prediabetes and concurrent HUA had significantly higher HOMA-IR values, suggesting more severe insulin resistance in this group. Moreover, individuals in the highest quartile of CRP/HDL-C had both higher HOMA-IR levels and greater risk of HUA compared with those in the lowest quartile, consistent with previous research. Obesity was highly prevalent among participants with diabetes or prediabetes. Those with coexisting HUA had significantly higher BMI, indicating a possible link between obesity and elevated SUA through two main pathways. One relates to lifestyle factors such as diets high in purine-rich meat, fat, and alcohol. The other involves adipokine regulation. Low adiponectin levels have been associated with both microvascular and macrovascular complications of diabetes, while leptin has shown variable associations with coronary heart disease in patients with type 2 diabetes (45). Notably, previous studies have reported an inverse relationship between SUA and adiponectin levels in hypertensive patients with MetS (46). Interestingly, the association between CRP/HDL-c and hyperuricemia was stronger in females than in males, with a significant interaction by gender. Interestingly, our gender-stratified analyses revealed that the association between the CRP/HDL ratio and HUA was stronger in females than in males. Notably, this association was most pronounced among women aged <50 years. Several biological and hormonal mechanisms may underlie this observation. Estrogen is known to exert anti-inflammatory effects and to enhance HDL-C levels, potentially leading to a more dynamic balance between pro- and anti-inflammatory processes in premenopausal women (47, 48). As a result, fluctuations in the CRP/HDL ratio may better reflect early metabolic disturbances in this population. Moreover, estrogen has been shown to facilitate uric acid excretion via renal pathways, and declining estrogen levels with age may attenuate this protective effect. Prior studies have similarly demonstrated sex-specific differences in inflammation and lipid metabolism, highlighting the need for gender- and age-specific risk stratification in metabolic disease (49). These findings suggest that the CRP/HDL ratio may serve as a particularly sensitive marker of hyperuricemia risk in younger women.

This study possesses several important strengths. First, it utilised data from a large-scale and well-established health management database at the Second Medical Centre of the Chinese PLA General Hospital, ensuring a substantial sample size with strong representativeness and generalizability. Second, potential confounding factors were rigorously controlled for during both the design and analytical phases, thereby enhancing the internal validity and reliability of the results. Third, restricted cubic spline (RCS) modelling was employed to examine potential non-linear associations between CRP/HDL-C and hyperuricemia, and subgroup analyses were conducted to assess the robustness and consistency of the findings across different population subgroups. Fourth, to account for the possibility that an elevated CRP/HDL-c ratio may arise from distinct individual alterations in CRP or HDL-c, we examined their associations with hyperuricemia separately. The consistent findings further supported the validity of CRP/HDL-c as a robust composite marker integrating inflammatory and lipid-related risk.

Nonetheless, several limitations should be acknowledged. First, the cross-sectional design of this study precludes causal inference, and the associations observed cannot establish temporal relationships. Longitudinal and interventional studies are warranted to elucidate underlying mechanisms. Second, although we adjusted for multiple covariates, residual confounding cannot be ruled out. For instance, we lacked data on the use of urate-lowering, lipid-lowering, and glucose-lowering medications, as well as key lifestyle factors such as alcohol consumption and dietary composition. Third, our study population exhibited relatively well-controlled glycemic levels, as indicated by low mean fasting glucose and HbA1c values. Future studies in populations with poorer glycemic control are needed to verify the generalizability of these findings. Fourth, although the CRP/HDL-c ratio functions as a composite marker integrating inflammation and lipid metabolism, it should be noted that a high ratio may result from elevated CRP, decreased HDL-c, or both. These scenarios may reflect different physiological mechanisms, and caution is warranted when interpreting the clinical implications of the ratio in isolation. Finally, although the CRP/HDL-c ratio was significantly associated with hyperuricemia in both males and females, we observed a stronger association in females (P for interaction = 0.031). Further subgroup analysis revealed that this association was particularly pronounced in females under 50 years old, with a significant interaction by age observed in women (P for interaction = 0.008). These findings underscore potential sex- and age-related heterogeneity, and suggest that this marker should be interpreted with consideration of demographic context and in conjunction with other clinical indicators.

## 5 Conclusion

We found a significant association between the CRP/HDL-c ratio and the risk of hyperuricemia in people with diabetes or prediabetes. Targeting inflammation and lipid metabolism to decrease this ratio may offer a potential strategy for risk assessment, prevention, and management of hyperuricemia in this population.

## Data Availability

The original contributions presented in the study are included in the article/**Supplementary Material**. Further inquiries can be directed to the corresponding authors.
