# Supplementary material for: Higher C-reactive protein to high-density lipoprotein cholesterol ratio is associated with hyperuricemia in diabetes and prediabetes: a cross-sectional study
Source: Front Endocrinol (Lausanne). 2025 Jun 27;16:1619370. doi: 10.3389/fendo.2025.1619370 (PMC12245703; doi:10.3389/fendo.2025.1619370)
Supplement: Supplementary file 1 [file DataSheet1.docx]

Supplementary Material

# Supplementary Figures and Tables

## Supplementary Figures

**
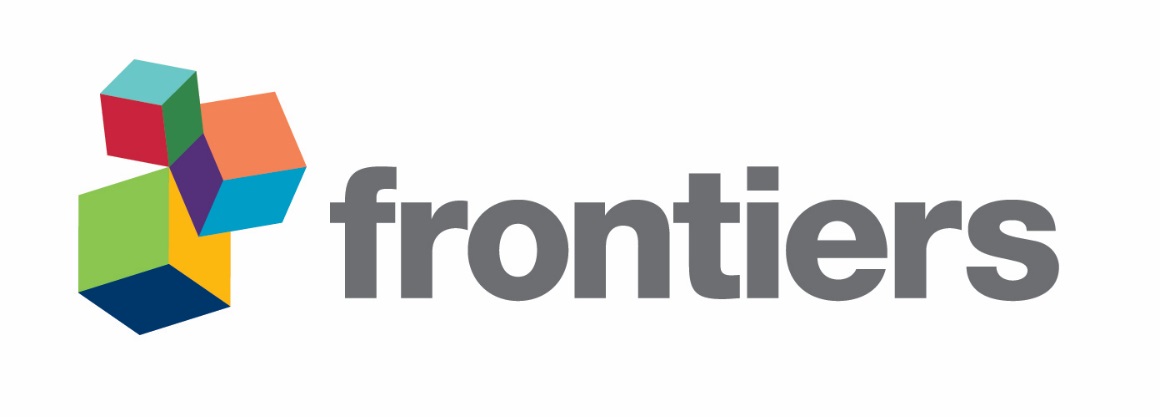
**

**Supplementary Figure 1.** Subgroup analysis of female participants by age group


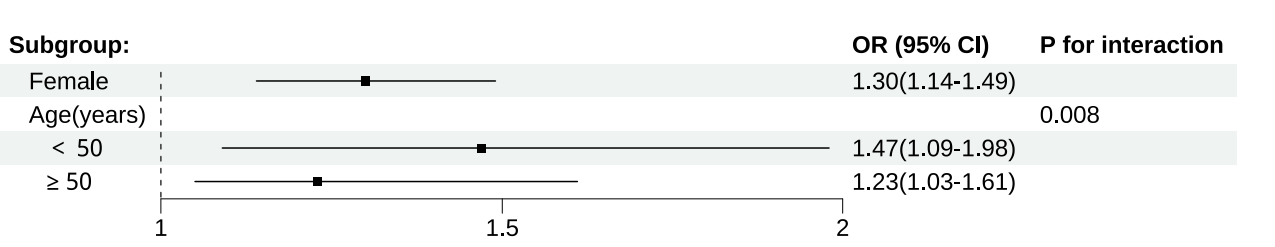


**Supplementary Figure 2.** RCS results


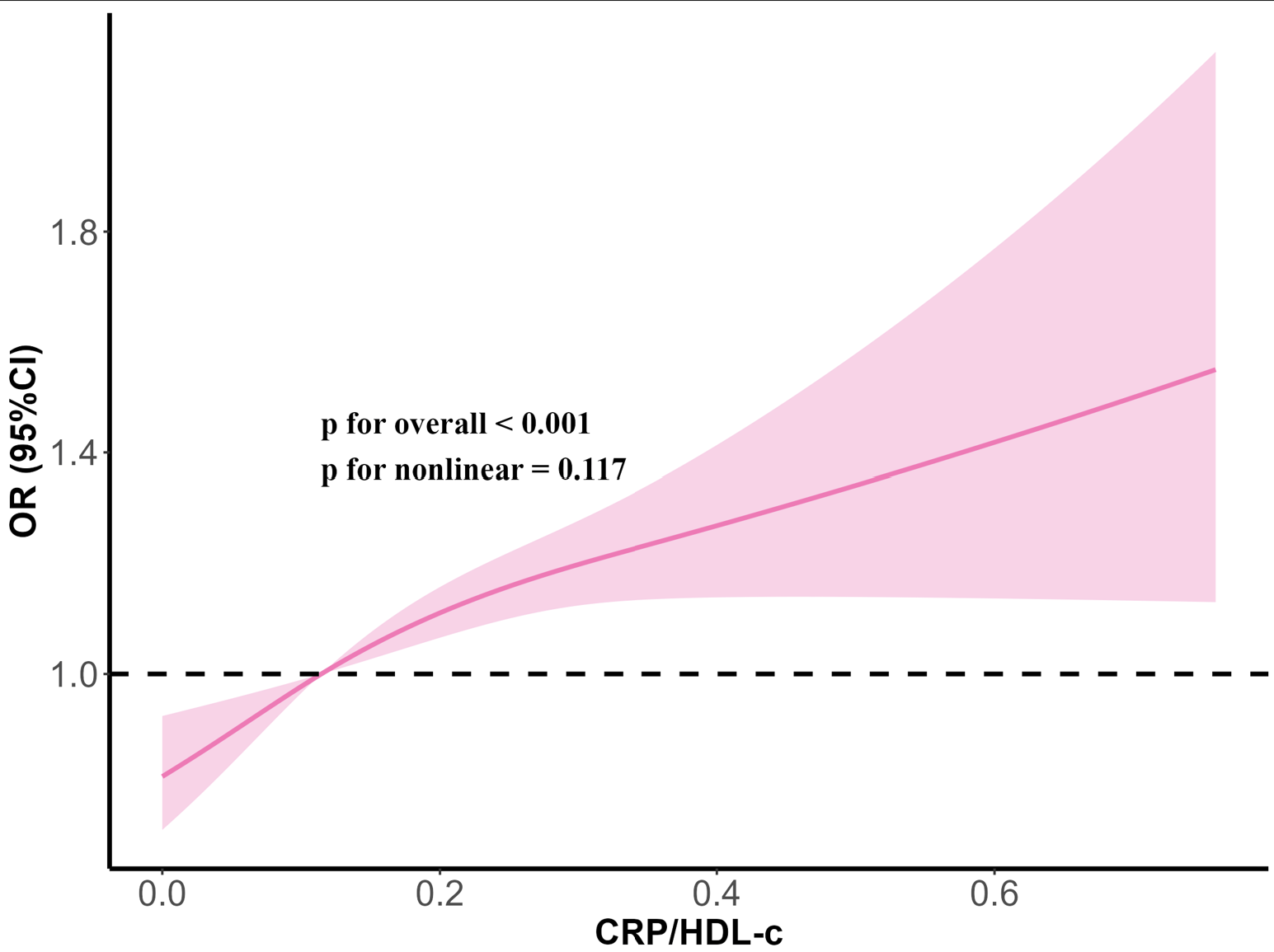


## Supplementary Tabels

**Supplementary Table 1** Multivariate logistic regression models of HUA.

| Characteristics | OR | 95%CI lower | 95%CI upper | P value |
| --- | --- | --- | --- | --- |
| CRP/HDL-c | 1.64 | 1.14 | 2.36 | 0.008 |
| Age (years) | 0.99 | 0.97 | 1.01 | 0.162 |
| Males (vs. females) | 0.88 | 0.58 | 1.34 | 0.561 |
| SBP (mmHg) | 1.01 | 1.00 | 1.01 | 0.009 |
| DBP (mmHg) | 1.00 | 1.00 | 1.01 | 0.424 |
| BMI (kg/m2) | 1.07 | 1.05 | 1.10 | <0.001 |
| Waist to hip ratio | 1.50 | 0.45 | 5.02 | 0.506 |
| ALT (U/L) | 1.00 | 0.99 | 1.00 | 0.469 |
| AST (U/L) | 1.02 | 1.01 | 1.04 | <0.001 |
| BUN (mmol/l) | 1.02 | 0.97 | 1.06 | 0.469 |
| Cr (umol/l) | 1.04 | 1.01 | 1.06 | 0.002 |
| eGFR (mL/min/1.73 m2) | 1.00 | 0.98 | 1.03 | 0.754 |
| FBG (mmol/l) | 0.92 | 0.85 | 1.00 | 0.046 |
| FCP (ng/ml) | 1.81 | 1.64 | 2.00 | <0.001 |
| FINS (mU/L) | 0.94 | 0.93 | 0.96 | <0.001 |
| TC (mmol/l) | 1.03 | 0.93 | 1.14 | 0.552 |
| TG (mmol/l) | 1.41 | 1.32 | 1.51 | <0.001 |
| LDL-c (mmol/l) | 1.10 | 0.99 | 1.22 | 0.085 |
| Drinkers (vs. no) | 0.87 | 0.79 | 0.97 | 0.009 |
| Diabetes (vs. no) | 0.72 | 0.62 | 0.83 | <0.001 |

**Supplementary Table 2** Multivariate linear regression models of SUA concentration.

| Characteristics | OR | 95%CI lower | 95%CI upper | P value |
| --- | --- | --- | --- | --- |
| CRP/HDL-c | 0.262 | 0.095 | 0.429 | 0.002 |
| Age (years) | -0.019 | -0.028 | -0.010 | <0.001 |
| Males (vs. females) | 0.943 | 0.769 | 1.12 | <0.001 |
| SBP (mmHg) | 0.002 | 0.000 | 0.003 | 0.096 |
| DBP (mmHg) | 0.003 | 0.000 | 0.006 | 0.061 |
| BMI (kg/m2) | 0.046 | 0.035 | 0.057 | <0.001 |
| Waist to hip ratio | 0.712 | 0.176 | 1.25 | 0.009 |
| ALT (U/L) | -0.001 | -0.004 | 0.003 | 0.715 |
| AST (U/L) | 0.016 | 0.010 | 0.022 | <0.001 |
| BUN (mmol/l) | 0.019 | 0.000, | 0.037 | 0.047 |
| Cr (umol/l) | 0.012 | 0.002 | 0.022 | 0.022 |
| eGFR (mL/min/1.73 m2) | -0.011 | -0.023 | 0.001 | 0.066 |
| FBG (mmol/l) | -0.045 | -0.079 | -0.011 | 0.009 |
| FCP (ng/ml) | 0.336 | 0.294 | 0.378 | <0.001 |
| FINS (mU/L) | -0.031 | -0.037 | -0.024 | <0.001 |
| TC (mmol/l) | 0.022 | -0.024 | 0.069 | 0.341 |
| TG (mmol/l) | 0.221 | 0.191 | 0.250 | <0.001 |
| LDL-c (mmol/l) | 0.046 | -0.002 | 0.094 | 0.061 |
| Drinkers (vs. no) | -0.036 | -0.080 | 0.008 | 0.111 |
| Diabetes (vs. no) | -0.287 | -0.350 | -0.223 | <0.001 |

**Supplementary Table 3** Logistic regression analysis of the association between CRP and HDL-c quartiles and the risk of HUA

| HUA | OR (95%CI) | P value |
| --- | --- | --- |
| CRP Categories |  |  |
| Quantile 1 | reference | reference |
| Quantile 2 | 1.00 (0.87–1.15) | 0.803 |
| Quantile 3 | 1.17 (1.01–1.34) | 0.031 |
| Quantile 4 | 1.26 (1.10–1.45) | 0.009 |
| HDL-c Categories |  |  |
| Quantile 1 | reference | reference |
| Quantile 2 | 1.04 (0.91–1.19) | 0.602 |
| Quantile 3 | 0.87 (0.73–0.98) | 0.045 |
| Quantile 4 | 0.83 (0.68–0.95) | 0.032 |

adjusted for age, gender, SBP, DBP, BMI, Waist to hip ratio, ALT, AST, BUN, Cr, eGFR, FBG, FCP, FINS, TC, TG, LDL-c, HbA1c, Drinkers, Hypertension.

| Variance inflation factor (VIF) | |
| --- | --- |
| Variables | VIF |
| CRP/HDL-c | 1.13 |
| Age (years) | 1.71 |
| Gender | 4.63 |
| SBP (mmHg) | 2.29 |
| DBP (mmHg) | 2.17 |
| BMI (kg/m2) | 2.31 |
| Waist to hip ratio | 2.77 |
| ALT (U/L) | 3.54 |
| AST (U/L) | 3.08 |
| BUN (mmol/l) | 1.16 |
| Cr (umol/l) | 3.47 |
| eGFR (mL/min/1.73 m2) | 5.23 |
| FBG (mmol/l) | 1.87 |
| FCP (ng/ml) | 3.82 |
| FINS (mU/L) | 3.77 |
| TC (mmol/l) | 4.32 |
| TG (mmol/l) | 1.82 |
| LDL-c (mmol/l) | 3.87 |
| Drinkers | 1.00 |
| Diabetes | 1.69 |
